# *Nkx2–5* Second Heart Field Target Gene *Ccdc117* Regulates DNA Metabolism and Proliferation

**DOI:** 10.1038/s41598-019-39078-5

**Published:** 2019-02-11

**Authors:** Anthony J. Horton, John Brooker, William S. Streitfeld, Meaghan E. Flessa, Balakrishnan Pillai, Raychel Simpson, Christopher D. Clark, Monika B. Gooz, Kimberly K. Sutton, Ann C. Foley, Kyu-Ho Lee

**Affiliations:** 1 Departments of Pediatrics and Obstetrics and Gynecology, Medical University of South Carolina, Charleston, SC 29425 USA; 2 Regenerative Medicine and Cell Biology Department, Medical University of South Carolina, Charleston, SC 29425 USA; 3 Department of Pharmaceutical and Biomedical Sciences, Medical University of South Carolina, Charleston, SC 29425 USA; 4 Bioengineering Department, Clemson University – MUSC, Charleston, SC 29425 USA

## Abstract

The cardiac transcription factor *Nkx2-5* is essential for normal outflow tract (OFT) and right ventricle (RV) development. *Nkx2-5*^−/−^ null mouse embryos display severe OFT and RV hypoplasia and a single ventricle phenotype due to decreased proliferation of Second Heart Field (SHF) cells, a pool of cardiac progenitors present in anterior pharyngeal arch mesoderm at mid-gestation. However, definition of the precise role of *Nkx2-5* in facilitating SHF expansion is incomplete. We have found that *Nkx2-5* positively and directly regulates a novel target gene, *Ccdc117*, in cells of the SHF at these stages. The nuclear/mitotic spindle associated protein Ccdc117 interacts with the MIP18/MMS19 cytoplasmic iron-sulfur (FeS) cluster assembly (CIA) complex, which transfers critical FeS clusters to several key enzymes with functions in DNA repair and replication. Loss of cellular Ccdc117 expression results in reduced proliferation rates associated with a delay at the G1-S transition, decreased rates of DNA synthesis, and unresolved DNA damage. These results implicate a novel role for *Nkx2-5* in the regulation of cell cycle events in the developing heart, through Ccdc117′s interaction with elements of the CIA pathway and the facilitation of DNA replication during SHF expansion.

## Introduction

While it has been shown that SHF proliferation is compromised in *Nkx2-5*^−/−^ null mouse embryos at E9.5, collective findings to date have provided limited insight as to how *Nkx2-5* expression directly controls SHF progenitor expansion and cell cycle events at these stages. Genetic studies in humans have associated *Nkx2-5* coding region point mutations with the familial and sporadic occurrence of congenital heart anomalies such as tetralogy of Fallot (ToF), ventricular septal defect (VSD), and atrial septal defect (ASD) similar to those observed in mouse knockout or hypomorphic expression mutants^1–6^. Molecular analysis of protein products resulting from *Nkx2-5* point mutations identified in these studies have demonstrated altered DNA binding affinity compared to wild-type, indicating that developmental pathology likely results from abnormal regulation of *Nkx2-5* target genes during heart development^7–10^. However, the studies of known pathways downstream of *Nkx2-5* in the SHF population have not addressed the mechanisms underlying direct control of cell cycle events.

**Figure 1:**
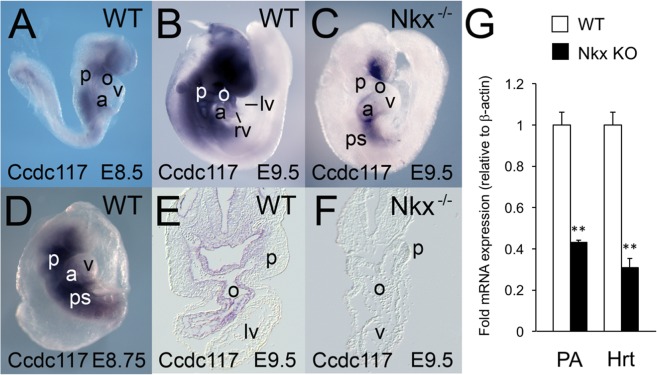
*Nkx2-5*-dependent expression of *Ccdc117* mRNA in the second heart field (SHF) and developing right heart. (**A**,**D**) *Ccdc117* mRNA expression (purple color) is first detected at E8.5–8.75 in pharyngeal arch and posterior splanchnic mesoderm near aortic and venous poles of the heart, respectively. Whole-mount (**B**) and section (**E**) views of *in situ* hybridization (ISH) for *Ccdc117* mRNA is shown in wild-type embryos at E9.5. *Ccdc117* expression was observed in the SHF-containing pharyngeal arch and the developing right ventricle, right atrium, and outflow tract. Whole-mount (**C**) and section (**F**) ISH results for *Ccdc117* mRNA in *Nkx2-5*^−/−^ null embryo at E9.5. *Ccdc117* mRNA expression is greatly reduced in the pharyngeal arch and developing OFT in mesodermal SHF progenitor cells and endodermal and endocardial populations. (**G**) qPCR for *Ccdc117* expression showing reduced mRNA expression in wild-type (white) vs. *Nkx2-5*^−/−^ null (black) SHF-containing pharyngeal arch (PA; approx. 40% of WT) or heart (Hrt; approx. 30% of WT). Results shown are the average and SEM of 3 independent samples each. **p < 0.01; by Student’s T-test). Abbreviations: p: pharyngeal arch; o: outflow tract; v: ventricle; a: atrium; rv: right ventricle; lv: left ventricle; ps: posterior splanchnic mesoderm.

We previously identified several novel direct target genes for *Nkx2-5* in the SHF region of mice during OFT development^11^. These included *Coiled-coil-domain-containing 117* (*Ccdc117*), which encodes an evolutionarily conserved protein of previously uncharacterized function. *In situ* hybridization (ISH) analysis of wild-type embryos first detected *Ccdc117* mRNA expression at E8.5 in pharyngeal arch regions, particularly in the first arch, and in developing OFT regions (Fig. 1A). Expression continued through E9.5 in the cardiac outflow tract and atria, and in SHF-containing pharyngeal arch with additional expression in lower craniofacial regions (Fig. 1B–D). Section analysis revealed *Ccdc117* mRNA expression in the developing outflow tract and SHF-associated pharyngeal mesoderm, with additional expression observed in pharyngeal endoderm, outflow tract endocardium and ventral neural tube populations (Fig. 1F). At later stages (E12.5 and above), *Ccdc117* mRNA expression became increasingly generalized in multiple tissues (data not shown).

To determine if *Ccdc117* expression in the SHF is dependent upon *Nkx2-5*, we assessed *Ccdc117* expression by *in situ* hybridization in E9.5 *Nkx2-5*^−/−^ null embryos. *Ccdc117* mRNA expression was greatly reduced in *Nkx2-5*-expressing OFT and pharyngeal endoderm and mesoderm, while being relatively retained in craniofacial mesoderm, atria and posterior splanchnic mesoderm. Interestingly, *Ccdc117* mRNA expression was also lost in OFT endocardium, where other investigators noted transient *Nkx2-5* expression in mice in a haemogenic endocardial lineage^12^. *Ccdc117* mRNA expression was also noted in dorsal pharyngeal mesoderm and ventral portions of the neural tube, indicating that *Nkx2-5* may regulate additional indirect and non-cell autonomous *Ccdc117* expression in these populations at this developmental stage. qRT-PCR assay confirmed the reduction of *Ccdc117* mRNA expression in SHF-containing pharyngeal arch of *Nkx2-5*^−/−^ knockout embryos as compared to wild-type (Fig. 1G).

These results were at odds with findings from our previous combinatorial mRNA expression microarray-based study, which predicted that *Ccdc117* was directly, but negatively, regulated by *Nkx2-5*^11^. This previous study was based on a re-analysis of publicly available, earlier generation expression microarray data derived from analysis of more extensive E9.5 cardiothoracic regions of wild-type and *Nkx2-5*^−/−^ mouse embryos than those used in our study, and included regions where we observed qualitatively less reduction of *Ccdc117* expression in the knockout (e.g., more anterior pharyngeal and more posterior lateral mesoderm (Fig. 1C)). These data were combined with data from later generation expression microarray analysis of differentiating P19 embryonal carcinoma cells. *Ccdc117* expression analysis may thus have been clouded by differences in microarray format, previous inclusion of embryonic regions with *Nkx2-5* independent regulation of *Ccdc117* mRNA expression, and confounding by *Ccdc117* expression in non-cardiac lineages present in P19 cultures^11^.

**Figure 2:**
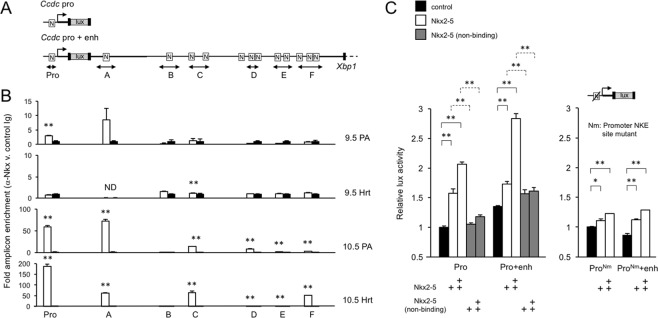
C*cdc117* regulatory regions are directly activated by Nkx2-5. (**A**) Diagram showing the composition of luciferase reporter constructs containing the *Ccdc117* 500–750 bp proximal promoter region/transcriptional start site, with or without an approximately 3 kb 3′flanking region shared with the neighboring *Xbp1* gene. Nkx2-5 consensus binding sites (NKE) are shown as boxed ‘N’s, and qPCR amplicons assayed in *in vivo* ChIP experiments are represented below. (**B**) Results from chromatin immunoprecipitation (ChIP) experiments using control and anti-Nkx2-5 antiserum, and chromatin from E9.5 and E10.5 SHF-containing pharyngeal arch (PA) and mouse hearts (Hrt) are shown below the schematic of NKE sites in the *Ccdc117* promoter region and the 3′ flanking region shown in A above. Results are expressed as relative fold enrichment of indicated amplicons overlapping the various NKEs obtained from Nkx2-5 vs. control antiserum and are representative of three independent experiments. **p < 0.02 by Student’s t-test. (**C**) Nkx2-5 activates *Ccdc117* promoter (pro) and promoter-enhancer (pro + enh) luciferase reporter activity in dose dependent fashion up to approximately 3-fold (activated (white) v. basal (black)). Activation is not observed with addition of the equivalent amount of expression plasmid for a non-DNA binding mutant isoform of Nkx2-5 (gray bars) (left panel). Alteration of the start site proximal NKE to an Nkx2-5 non-binding consensus similarly greatly reduces activation of promoter (Pro) and promoter-enhancer (Pro + enh) reporter activity (right panel). Results are shown as average and SEM of duplicate samples and are representative of three independent experiments. *p < 0.05, **p < 0.01 by Student’s T-test.

Our previous study identified an Nkx2-5 binding consensus sequence (NKE) in the proximal promoter region of *Ccdc117*^11^. Further analysis of *Ccdc117* genomic flanking regions identified multiple predicted NKEs in the 3′ untranslated region (UTR) of *Ccdc117* shared with its immediate 3′ neighbor, *Xbp1*^13^ (Fig. 2A). To evaluate the likelihood of direct regulation of *Ccdc117* by *Nkx2-5 in vivo*, we examined promoter and regulatory region occupancy by Nkx2-5 in developing embryos using chromatin immunoprecipitation (ChIP) analysis. ChIP assay using an antibody specific for Nkx2-5 detected significant interaction of Nkx2-5 protein with the *Ccdc117* promoter region and the most promoter proximal 3′ Nkx2-5 binding site *in vivo* in E9.5-E10.5 SHF-containing PA (Fig. 2B). Interestingly, the proximal 3′ Nkx2-5 binding region was also identified by a ChIP-seq study performed in the HL-1 atrial cardiac cell line using biotinylated Nkx2-5^14^. Additional interactions were detected with more distal Nkx2-5 binding sites at E10.5. While significant Nkx2-5 binding to predicted NKE sites was largely not detected in E9.5 heart, significant interactions were detected at E10.5. These data are all consistent with the evolving direct and positive regulation of *Ccdc117* by Nkx2-5 in developing SHF and heart.

Cell reporter assays confirmed that Nkx2-5 expression directly activates a *Ccdc117*-based promoter-enhancer construct incorporating the 3′ consensus Nkx2-5 binding sites and the *Ccdc117* proximal promoter in dose-dependent fashion (Fig. 2C, left). This activation is significantly weaker in the absence of the NKE-containing 3′ enhancer region (Fig. 2C, left), and was largely abrogated by alternative expression of a mutant isoform of Nkx2-5 (Ile183→Pro) known to lack binding activity to NKE consensus sites^7,15^. Additionally, alteration of the transcriptional start site-proximal NKE identified in our past study, that also shows evidence of significant Nkx2-5 interaction *in vivo* (Fig. 2B), to an Nkx2-5 non-binding sequence was sufficient to greatly inhibit activation of both promoter and promoter-enhancer reporters (Fig. 2C, right)^11,15^.

The *Ccdc117* gene encodes a predicted 277 AA/30.4 kDa protein sequence that is strongly conserved in placental mammals and more variably conserved in other vertebrates. While its predicted amino acid sequence contains no other recognizable conserved functional domains, its name derives from a conserved region (AA 141-170 in mouse) highly likely to adopt a coiled-coil structure (Supplemental Fig. 1, red box). Since coiled-coil domains frequently mediate protein-protein interactions, we performed a yeast two-hybrid screen to identify Ccdc117 binding partners and to gain insight into its involvement in functional pathways.

Using a bait consisting of a fusion between the full length human Ccdc117 protein and the yeast GAL4 DNA binding domain, we screened approximately 1 × 10^8^ clones from a normalized “universal” mouse cDNA library broadly representative of multiple tissues. We obtained 196 independent clones, slightly over half of which (103) represented multiple isolates (n = 2–10) of identical or overlapping genes, indicating a high likelihood of having achieved saturation. The 61 unique candidates represented several functional classes of proteins found to exhibit nuclear and mitotic spindle localization, metal binding, and to function in intracellular membrane vesicle trafficking, protein synthesis, folding, degradation and transport, and mitochondrial activity.

**Figure 3:**
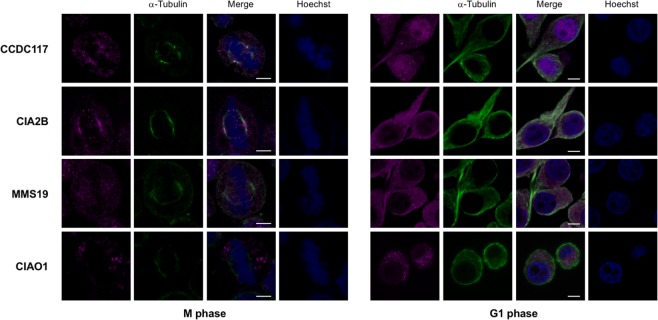
Ccdc117 cellular localization and interaction with the MMS19 CIA complex. Immunofluorescence (IF) confocal images of M-phase (*left*) and interphase (G1) (*right*) HeLa cells comparing immunolocalization of Ccdc117 to that of MMS19 CIA complex components CIA2B, MMS19, and CIAO1 (magenta) vs. mitotic spindle (α-tubulin, green) and metaphase chromosomes and nuclei (Hoechst 3358, blue). Scale bar: 5 um.

One candidate interacting protein, MIP18/Fam96B/CIA2B (hereafter called CIA2B), is a component of the MIP18/MMS19 (hereafter referred to as MMS19) cellular iron sulfur cluster assembly (CIA) targeting complex^16^. The MMS19 complex, containing MMS19, CIA2B, and CIAO1, is characteristically localized to the mitotic spindle at M-phase in HeLa and other mammalian cells, and functions in the cytosolic transport and transfer of elemental iron sulfur (FeS) clusters originally assembled in mitochondria^16,17^. While early embryonic expression of *Ccdc117* is limited to SHF and related regions (Fig. 1), *Ccdc117* is expressed in multiple adult tissues and cell lines including HeLa cells^18^. As shown in Fig. 3, immunofluorescent (IF) localization assay in M-phase HeLa cells detects Ccdc117 localization to the mitotic spindle similar that of other MMS19 complex members (Fig. 3, top row, left) (see also quantitative localization data in Supplementary Materials). In interphase (G1) HeLa cells, Ccdc117 protein is detected both in nuclear and in punctate cytoplasmic compartments (Fig. 3, top row, right). This localization is similar to that observed for *MMS19* complex members, with the exception of CIAO1, which shows little or no nuclear localization in G1 (Fig. 3, bottom rows).

**Figure 4:**
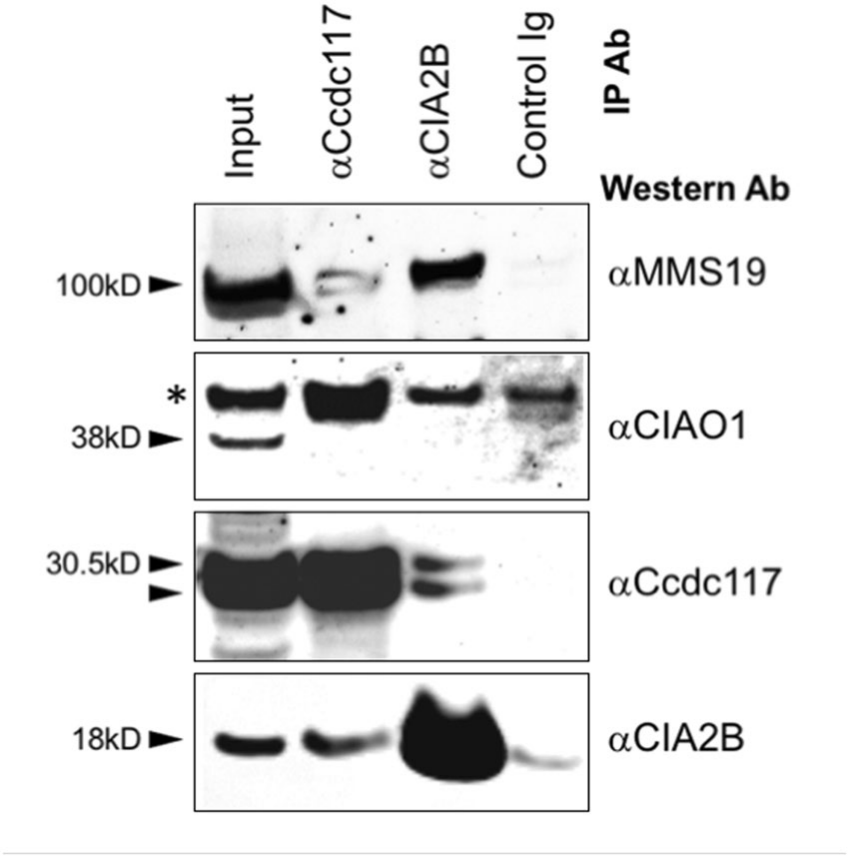
Reciprocal co-IP experiments detect interaction of Ccdc117 with CIA2B, and interaction of both with MMS19, but not CIAO1 in cycling HeLa cell extracts. Input lysate and IP samples by antibody are shown vertically at tops of lanes; primary Ab used for Western are shown to right. MW are shown to left. * indicates a non-specific band detected by anti-CIAO1 antibody.

Co-immunoprecipitation (Co-IP) assays of cycling native HeLa cell extracts using antibodies specific for *Ccdc117* and *CIA2B* independently confirmed the capacity for physical interaction between these two proteins. IP with anti-Ccdc117 antibody also retrieved CIA2B (Fig. 4, row 4, second column), while IP with anti-*CIA2B* antibody likewise recovered Ccdc117 (Fig. 4, row 3, third column). IP with either anti-Ccdc117 or anti-CIA2B antibody also recovered MMS19 in these extracts (Fig. 4, row 1). The interaction with MMS19 appeared to be somewhat weaker in the case of anti-Ccdc117 IP, potentially indicating a more indirect association between Ccdc117 and MMS19. Interestingly, CIAO1, which lacked nuclear localization in interphase cells, was not recovered by IP with either anti-Ccdc117 or anti-CIA2B (Fig. 4, row 2).

The MMS19 complex receives iron-sulfur (FeS) clusters from other cytosolic transfer and chaperone proteins and targets their final transfer to several key enzymes regulating DNA synthesis and repair^19^. These target enzymes include the XPD family of DNA repair helicases, DNA primase, and the catalytic subunit of class B DNA polymerases α, δ and ε. Interaction of these target apoproteins with FeS has been shown to be functionally important, as the DNA synthesizing or repair activities of mutant isoforms unable to bind FeS are severely compromised^17,19–21^. Loss of MMS19 activity similarly affected DNA metabolism: deletion mutation of *MMS19*, and siRNA knockdown of MMS19 resulted in defects in nucleotide excision repair and related defects in chromosomal segregation and nuclear morphology^16,22^. We reasoned that if Ccdc117 interaction played a functional role with respect to MMS19 FeS targeting activity, loss of Ccdc117 expression might compromise the function of MMS19 target enzymes and similarly inhibit DNA metabolism, resulting in decreased cell proliferation rates. We therefore tested the potential for functional interaction of Ccdc117 with the MMS19 CIA targeting complex through loss-of-function siRNA knockdown experiments.

**Figure 5:**
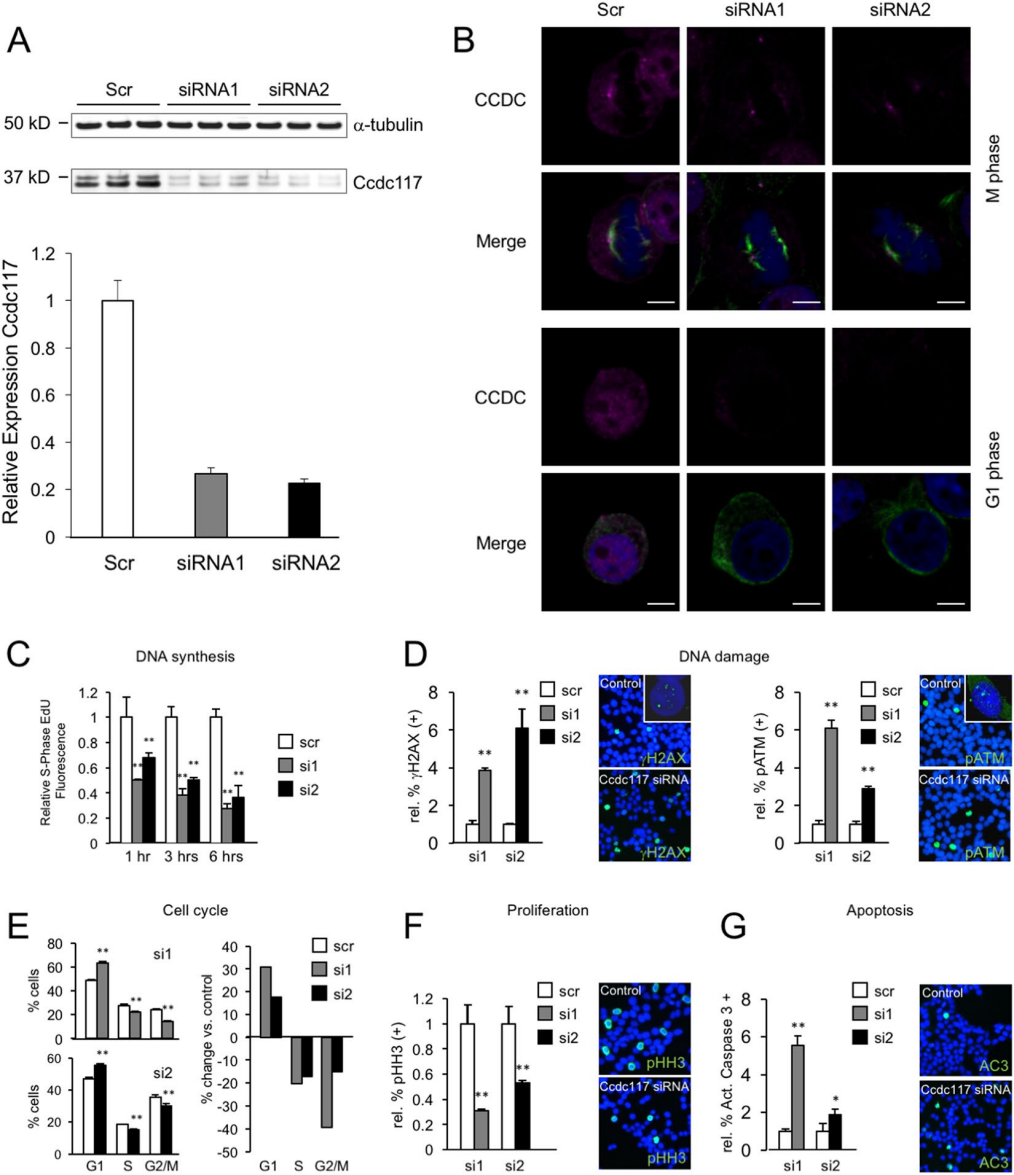
Ccdc117 knockdown results in decreased rates of DNA synthesis, increased DNA damage, inhibition of cell cycle progression and reduced cell proliferation. (**A**) Knockdown with two independent siRNAs results in approx. 80% reduction in Ccdc117 protein expression relative to a-tubulin in triplicate knockdown (KD) experiments. (**B**) IF for Ccdc117 confirms specific loss of spindle-associated, cytoplasmic and nuclear fluorescence signal following anti-Ccdc117 knockdown. (C) siRNA-mediated KD of Ccdc117 expression in HeLa cells results in progressive and significant decreases (40–50% at 1 hr → 65–75% at 6 hrs) in S-phase DNA synthesis rates as detected by EdU incorporation. (D) Increased positivity for nuclear γH2AX and pATM (right panels; insets: close-up confocal images showing punctate nuclear staining for γH2AX and pATM) following Ccdc117 knockdown with two distinct siRNAs, expressed as relative change in percent positivity compared to scrambled siRNA control (γH2AX: siRNA1: 3.85 ± 0.14-fold; siRNA2: 6.10 ± 1.0 fold. pATM: siRNA1: 6.11 ± 0.42-fold; siRNA2: 2.89 + 0.14-fold. (E) apparent cell cycle delay at G1/S transition following Ccdc117 knockdown with two distinct siRNAs, expressed as percent of cells at each phase following siRNA (siRNA1, gray; siRNA2, black) as compared to scrambled control (Scr, white) on the left, and expressed as change in percent of cells at each cell cycle phase on right: G1: siRNA1: +24.3%; siRNA2: +24.7%; S: siRNA1: −24.7%; siRNA2: −20.3%; G2/M: siRNA1: −42.6; siRNA2: −24.0%. (F) Decreased proliferation rate as determined by relative change in percent phosphohistone H3 (PHH3; right panels) positivity following Ccdc117 knockdown with two distinct siRNAs: siRNA1: 0.31 ± 0.02-fold; siRNA2: 0.52 ± 0.02-fold. (G) increased rates of apoptosis assayed by activated caspase 3 (AC3, right panels) positivity following Ccdc117 knockdown with two distinct siRNAs expressed as relative change in percent positivity: siRNA1: 5.53 ± 0.50-fold; siRNA2: 1.86 ± 0.33-fold. **p < 0.02.

Using two independent siRNA molecules targeting Ccdc117, we were able to achieve approximately 80% reduction in Ccdc117 protein expression in transiently transfected HeLa cells as compared to a scrambled control (Fig. 5A). IF detected an associated decrease in mitotic spindle positivity for Ccdc117 at M-phase, and of nuclear and cytoplasmic positivity at G1 phase (Fig. 5B). siRNA knockdown of Ccdc117 protein expression resulted in decreased rates of S-phase DNA synthesis, as evinced by progressively reduced levels of EdU incorporation over time (Fig. 5C). IF detected an associated increase in nuclear positivity for two sensitive and independent markers of DNA damage, nuclear γ−phosphohistone H2AX (γ-H2AX)^23^, and phosphorylated ATM checkpoint kinase (pATM)^24^, in cells subject to anti-*Ccdc117* siRNA knockdown as compared to scrambled control (Fig. 5D).

As a possible consequence of reduced DNA synthesis rates and/or increased unresolved DNA damage, Ccdc117 siRNA knockdown HeLa cells exhibited an apparent delay of cell cycle progression at the G1 – S or intra S phases. Cell cycle profiling by flow cytometric analysis showed an increase in the proportion of cells in G0/G1 phase, with corresponding decreases in the percentage of cells in S and G2/M phases as compared to control scrambled siRNA (Fig. 5E). Immunoassay for the fraction of cells in mitosis or M phase using positivity for phospho-histone H3 (pHH3) independently confirmed a decrease in the proportion of cells in M-phase (Fig. 5F**)**. As a potential consequence of prolonged unresolved DNA damage, we observed a variable increase in the rate of apoptosis as measured by immunostaining for activated caspase 3 in Ccdc117 siRNA knockdown cells as compared to control (Fig. 5G).

**Figure 6:**
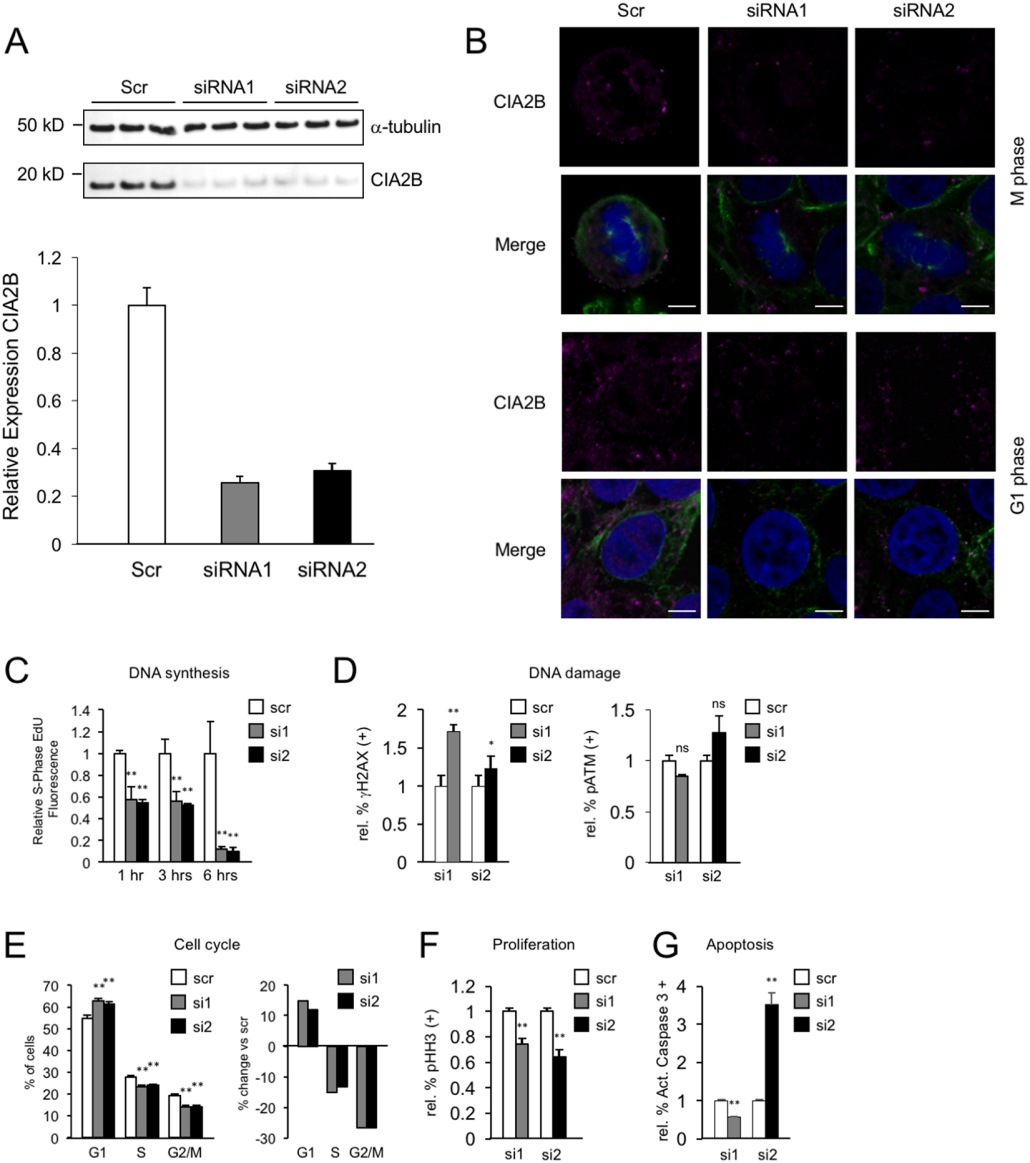
CIA2B knockdown results in decreased rates of DNA synthesis, increased DNA damage, inhibition of cell cycle progression, and reduced cell proliferation. (**A**) Knockdown with two independent siRNAs results in approx. 70–75% reduction in CIA2B protein expression relative to α-tubulin in triplicate knockdown (KD) experiments. (**B**) IF for CIA2B confirms specific loss of spindle-associated, cytoplasmic, and nuclear fluorescence signal following anti-CIA2B knockdown. (C) siRNA-mediated KD of CIA2B expression in HeLa cells results in progressive and significant decreases (40% at 1 hr → 90% at 6 hrs) in S-phase DNA synthesis rates as detected by EdU incorporation. (D) Changes in positivity for nuclear γH2AX and pATM following CIA2B knockdown with two distinct siRNAs, expressed as relative change in percent positivity compared to scrambled siRNA control (γH2AX: siRNA1: 1.72 ± 0.09-fold; siRNA2: 1.23 ± 0.17-fold. pATM: siRNA1: 0.85 ± 0.01-fold; siRNA2: 1.28 ± 0.16-fold. (E) Apparent cell cycle delay at G1/S transition following CIA2B knockdown with two distinct siRNAs, expressed as percent of cells at each phase following siRNA (siRNA1, gray; siRNA2, black) as compared to scrambled control (Scr, white) on the left, and expressed as percent change in proportion of cells at each cell cycle phase on right: G1: siRNA1: + 14.8%; siRNA2: +12.0%; S: siRNA1: −15.0%; siRNA2: −13.3%; G2/M: siRNA1: −26.8%; siRNA2: −26.6%. (F) Decreased proliferation rate as determined by relative change in percent PHH3 positivity following CIA2B knockdown with two distinct siRNAs: siRNA1: 0.74 ± 0.04-fold; siRNA2: 0.64 ± 0.06-fold. (G) altered rates of apoptosis assayed by AC3 positivity following CIA2B knockdown with two distinct siRNAs expressed as relative change in percent positivity (siRNA1: 0.57 ± 0.01-fold; siRNA2: 3.51 ± 0.31-fold). **p < 0.02; *p < 0.05; ns = not significant.

Knockdown of protein expression of CIA2B, the Ccdc117-interacting component of the MMS19 targeting complex, in HeLa cells similarly resulted in parallel changes in the rate of DNA synthesis, DNA damage repair and cell cycle progression. Using two independent siRNAs, we were able to achieve 70–75% knockdown of CIA2B protein expression level (Fig. 6A), with associated loss of mitotic spindle-associated IF signal at M-phase and nuclear and cytoplasmic IF signal at G1 phase (Fig. 6B). As was observed with Ccdc117 knockdown, siRNA knockdown of CIA2B expression in HeLa cells resulted in substantially decreased rates of DNA synthesis at S-phase (Fig. 6C). CIA2B knockdown also resulted in evidence of increased DNA damage (Fig. 6D). Interestingly, while increases in DNA damage markers were all qualitatively similar to those found following Ccdc117 knockdown, they were of lesser magnitude and consistency, and increases in apoptosis were similarly less consistent (Fig. 6G). Overall, CIA2B knockdown also resulted in apparent cell cycle delay at the G1-S transition (Fig. 6E), and decreased percentages of cells found in M-phase (Fig. 6F). While these differences in knockdown phenotype might be explained by a slightly lesser degree of knockdown achieved by siRNA (compare Figs 5A and 6A), they might also reflect an incomplete or partial overlap of Ccdc117 vs CIA2B interactions with the multiple and diverse CIA complexes characterized to date^17,21^. Specifically, Ccdc117 interaction with additional partners other than CIA2B in CIA assembly and/or other metabolic pathways may account for the stronger or more exaggerated phenotypes observed following its knockdown as compared to CIA2B knockdown.

**Figure 7:**
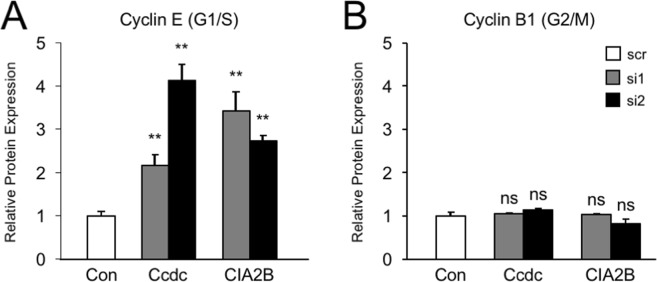
siRNA knockdown of Ccdc117 and CIA2B results in altered G1/S and G2/M cyclin expression. (**A**) siRNA (gray and black bars) results in significantly elevated expression of nuclear Cyclin E (Ccdc117: +2.17 ± 0.23-fold (siRNA1), +4.13 ± 0.37-fold (siRNA2); CIA2B: 3.42 ± 0.44-fold (siRNA1), +2.73 ± 0.13-fold (siRNA2). (**B**) Cyclin B1 expression is unchanged following siRNA: (Ccdc117: +1.06 ± 0.02-fold (siRNA1), +1.14 ± 0.04-fold (siRNA2); CIA2B: +1.03 ± 0.02-fold (siRNA1), +0.83 ± 0.10-fold (siRNA2). **p < 0.02; ns: not significant.

As a second and independent measure of changes in cell cycle progression, we found that siRNA knockdown of either Ccdc117 or CIA2B in cycling HeLa cells resulted in significantly increased protein levels of nuclear Cyclin E, consistent with an inhibition of cell cycle progression at G1/S (Fig. 7A). By contrast, we detected no evidence of delay at the G2-M transition, as no significant elevation of M-phase specific nuclear CyclinB1 protein levels was observed (Fig. 7B). These data are additionally consistent with a role for Ccdc117-CIA2B interaction primarily at the G1/S transition or in early S-phase.

**Figure 8:**
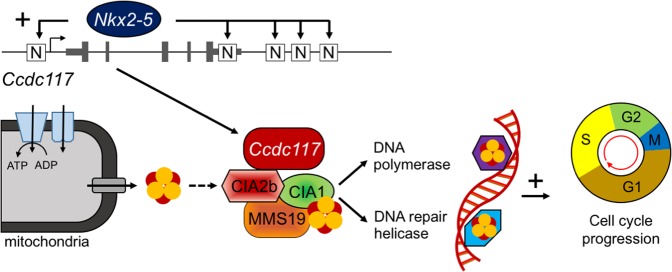
Summary model of *Ccdc117* facilitation of cell proliferation downstream of *Nkx2-5*.

Taken together, these data support a functional interaction between Ccdc117 and MMS19 in facilitating DNA repair, cell cycle progression, and cell proliferation. Accordingly, they suggest a novel mechanistic hypothesis to explain the cardiac proliferation defects observed in *Nkx2-5* knockout whereby loss of Ccdc117 expression in SHF progenitors leads to cell cycle delay, loss of proliferation, and OFT/right heart morphogenic defects (Fig. 8).

*Nkx2-5* plays a key role in the development of the right heart and OFT through its participation in gene programs regulating the balance between proliferation and differentiation of SHF progenitors. The regulation of this process is dependent upon the overall balance between competing pro-proliferative signals such as FGF8^25–30^, Shh^31,32^ and Wnt^33^, and anti-proliferative/pro-differentiation signals, principally BMP2^27,28^. At the level of transcriptional control and like several other cardiac-specifying genes, *Nkx2-5* expression in the SHF is mediated by combinatorial interactions between other lineage-specific TFs and transcriptional co-factors and transcriptional mediators of developmental signaling; e.g., canonical Smad pathways activated by BMP signaling^15,34–36^. The combinatorial nature of such embryonic cardiac transcriptional activation is facilitated by extensive protein-protein interactions between cardiac TFs^15,37–39^ and likely culminates in the recruitment of EP300 histone acetyltransferase activity, which has been found to be highly associated many embryonic cardiac regulatory regions^15,40,41^. Beyond this current study, *Ccdc117* flanking or regulatory regions have yet to be implicated as targets of other cardiogenic TFs or related factors. However, examination of the ENCODE compendium identifies interactions in multiple cell types between *Ccdc117* flanking and intronic regions with potentially cardiogenic regulatory factors; e.g., GATA zinc finger proteins, SRF, Mef2c and YY1, whose potential contribution to cardiac or SHF regulation of *Ccdc117* have yet to be determined^42^.

The decreased proliferation of SHF progenitors observed in *Nkx2-5* knockout mouse embryos has previously been associated with attenuation of Wnt-mediated proliferation signals due to loss of *Nkx2-5*-driven Rspondin3 expression^33^, and with the de-repression of BMP2 expression and associated signaling in SHF and outflow tract cells^5,33^. However, while other cardiac developmental transcription factors have been directly related to changes in downstream expression cell cycle regulators, principally the D-type cyclins and Cdks^43,44^, such a direct link to cell cycle control *per se* has been previously lacking for *Nkx2-5*.

Control of embryonic cardiac cell proliferation is a critical mediator of heart development, and prior studies have documented high rates of proliferation and rapid cell cycle progression and regionally variable cell cycle lengths with during SHF stages^45^. These rapid rates of cell proliferation would render cardiac progenitors particularly vulnerable to replication stress, by placing an increased demand on DNA synthesis rates and DNA proofreading and repair functions necessary for accurate replication of the genome. The recent discovery that mitochondrially-generated FeS clusters are trafficked into cytosolic and nuclear compartments where they act as functionally important co-factors for DNA metabolizing enzymes provided a seminal link between cell proliferation, and cellular bioenergetics, iron uptake and redox balance. It also provided mechanistic insight into why mutations of FeS assembly pathway components are associated with developmental defects and diseases involving multiple cell types^19,46–49^. While *Nkx2-5* apparently plays a key role in regulating *Ccdc117* expression in SHF progenitors early in heart development, other mechanisms may control *Ccdc117* expression in multiple other surrounding tissues later in embryonic development, as we observed much more general expression in multiple embryonic regions after mid-gestation.

Similarly, the extent and spatiotemporal specificity of Ccdc117 co-localization and functional interaction with members of the MMS19 targeting complex and other CIA pathway components remain to be determined. For example, the relationship of the striking similarity in mitotic spindle vs. nuclear and cytoplasmic localization of Ccdc117 and MMS19 components to the observed effects of *Ccdc117* and *CIA2B* LOF at G1 and early S remains unclear. Future investigations will be directed toward a better understanding of the significance of compartment-specific association to functional interactions leading to FeS assembly and transfer.

Overall, the identification of *Ccdc117* as both a direct downstream target of *Nkx2-5* regulation, and as a functional interacting partner of the MMS19 CIA targeting complex provides a novel and insightful link between metabolic state and the cardiac morphogenic pathways underlying CHD via the harnessing of FeS synthesis, assembly, and targeting pathways regulating cell proliferation.

## Methods

### Animal usage (mouse)

All mouse experiments were carried out under an animal protocol approved by the Institution Animal Care and Use Committee (IACUC) at the Medical University of South Carolina in compliance with the approved guidelines and methods.

### *In situ* mRNA hybridization

E9.5 wild-type and *Nkx2-5*^−/−^ null embryos were harvested by dissection from timed pregnancies, separated from extra embryonic tissues, fixed in 4% paraformaldehyde overnight and dehydrated through an alcohol series. Antisense and control sense digoxigenin-labeled riboprobes were reverse transcribed from a linearized full-length *Ccdc117* cDNA clone in pCMV-SPORT6 (Thermo Scientific/Open Biosystems, Huntsville, AL) using the DIG RNA Labeling Kit Sp6/T7 (Roche, Indianapolis, IN). Hybridization was performed at 63 °C according to previously established protocols (Hogan, 1994 and Barth *el al*.^11^). For section analysis, wholemount stained embryos were dehydrated, Paraplast embedded, sectioned and mounted in Permount (Sigma, St. Louis MO) prior to brightfield digital photography on a Zeiss Axioimager M2.

### RNA Purification, cDNA Synthesis, and Quantitative RT-PCR

Tissue samples of the SHF-containing pharyngeal arch and hearts of wild-type and *Nkx2-5*^−/−^ null embryos were collected by dissection at E9.5 and RNA prepared from tissue samples by Trizol extraction and additional purification using the RNeasy Mini kit (Qiagen, Valencia, CA). RNA samples were reverse transcribed into cDNA using the iScript cDNA Synthesis Kit (Biorad, Hercules, CA). Quantitative RT-PCR was performed using the iQ SYBR green/iCycler amplification system (Bio-Rad, Hercules, CA) and gene-specific oligonucleotide primers for *Ccdc117* and compared to control amplifications using β-actin primers^11^. Quantitative RT-PCR assays were performed in triplicates, and results shown are averages and standard deviations of three independent wild-type and three *Nkx2-5*^−/−^ null pharyngeal arch and heart explants. Relative expression levels were quantified using the ΔΔC(t) statistical method as previously described (Livak and Schmittgen 2001, Allen *et al*. 2009). Statistical significance for triplicate qPCR results were calculated using the 2-tailed Student’s t-test.

### Reporter Constructs and Promoter Assay

Promoter-enhancer plasmid reporter constructs were made from a reporter plasmid based on pHsp68 lacZ and luciferase coding sequences from pGL3 basic (Promega, Madison, WI) (Lee *et al*.^34^). A 747 bp fragment of the *Ccdc117* 5′ flanking region and transcriptional start site was amplified and ligated 5′ to the luciferase coding region to create the *Ccdc117*-pro-lux promoter reporter plasmid. The *Ccdc117*-pro-enh-lux reporter plasmid was created by the additional 3′ directional insertion of an approximately 3 kb genomic region encompassing the 3′ UTR of the mouse *Ccdc117* gene shared with the neighboring *Xbp1* gene. For the Nm promoter NKE site mutant assays, a 747 bp fragment where the –(520-527) bp (relative to the translational start site) NKE sequence was altered from TGAAGTG to a non-binding TGAcgaa sequence^15^ was synthesized (IDTDNA), confirmed by Sanger sequencing, and cloned via 5′ XhoI and 3′ NcoI sites into recipient promoter and promoter-enhancer luciferase reporter minigenes for assay.

Reporter constructs were assayed in P19CL6 cells as previously described (Lee *et al*.^34^, Clark *et al*.^15^). 0.025 µg of reporter plasmid per 24-well plate well was co-transfected with varying amounts of pCS2-*Nkx2-5* expression plasmid (0.0, 0.05 or 0.1 µg/well) (Clark *et al*.^15^), or pCS2-*Nkx2-5* Ile(183) → Pro^50^, and 0.005 µg pCMV-renilla or pTK-renilla control plasmids. Following 18–20 hr incubation cells were lysed and subject to dual luciferase assay (Promega) according to manufacturer protocols. Results were assayed in duplicate wells and are expressed as fold activation over control reporter alone normalized to renilla luciferase activity. They are representative of three independent trials each. Statistical significance was calculated using 2-tailed Student’s t-test.

### *In vivo* Chromatin Immunoprecipitation (ChIP)

Chromatin was extracted from approximately 50 wild-type FVB E9.5 mouse hearts and assayed by ChIP according to established protocols (Barth *et al*.^11^) using either Nkx2-5 anti-serum or control pre-immune anti-serum^51^. Immunoprecipitated chromatin was assayed by qPCR using primers amplifying *Ccdc117* 3′ genomic flanking regions encompassing Nkx2-5 binding consensus sites (NKEs). qPCR results are expressed as fold enrichment comparing amplicons recovered using anti-*Nkx2-5* antibody vs. control antiserum based upon ΔΔC(t) calculations compared to control non-immune anti-serum, and are representative of three independent trials^11^.

### Yeast two-hybrid screening

Yeast two-hybrid screening was performed with the Matchmaker Gold kit (Clontech) and a normalized Universal Mouse cDNA prey library (Clontech #630482) according to manufacturer instructions. Briefly, a bait construct was engineered by inserting the full-length coding region of mouse *Ccdc117* amplified from cloned cDNA (Open Biosystems) to create a fusion protein with the yeast Gal4 activator domain (AD). The *Ccdc117*-Gal4AD expression plasmid was used to create a stably transformed cell line in Y2H Gold yeast. Following library transfection of approximately 1 × 10^8^ bait clone recombinants via mating a pre-transformed library strain, 452 independent transformants were manually selected and further screened for β-galactosidase expression to yield resistant colonies representing 195 identifiable cDNA coding regions. Of these, over half were multiply represented in independent isolates, indicating a high likelihood of saturation.

### Immunofluorescence (IF) assays

HeLa cells were seeded onto gelatin and fibronectin coated coverslips at an initial seeding density of approximately 1 × 10^6^ cells per well. For immunolocalization studies, cells were fixed in 4% paraformaldehyde and permeabilized with 0.05% Triton X-100 in PBS. Primary antibody incubation was followed by staining with Cy5-conjugated secondary antibody staining and DNA counterstaining with Hoechst 33258 (Life Technologies). For proliferation (phosphohistone H3), apoptosis (activated caspase 3), and DNA damage (γ-phosphohistone H2AX) staining, coverslip mounted cells were rinsed in PBS and fixed in 95% methanol/5% glacial acetic acid, followed by Triton X-100 permeabilization as above. Samples were imaged with a Zeiss LSM 880 NLO Axioobserver inverted laser scanning confocal/multiphoton microscope (Thornwood, NY) using a 63 × 1.4 N.A. Plan-Apochromat oil immersion DIC lens. Fluorescence of DAPI, TRITC and Cy5 were excited at 405 nm, 561 nm, and 633 nm respectively and detected at 410–547 nm, 565–650 nm, and 638–759 nm respectively at a 1-Airy-unit-diameter pinhole. Images were processed using Zeiss Zen software.

For DNA damage (γH2AX, pATM), proliferation (pHH3) and apoptosis (AC3) assays following siRNA knockdown, cells were fixed and permeabilized in ice cold 95% ethanol/5% acetic acid prior to primary antibody and Alexa 433 secondary antibody staining. Stained cells were imaged with a Zeiss Axio Imager M2. For DNA damage assays, nuclei were scored as being positive for γH2AX and nuclear pATM if ≥5 punctate foci were observed. Percentage of positive nuclei were based on triplicate counts of ≥500 nuclei and are representative of triplicate experiments.

### siRNA knockdown

Approximately 1 × 10^6^ HeLa cells were seeded in 6-well plate wells prior to transfection with 10 nM siRNA oligomers (OriGene) targeting the human *Ccdc117* gene (SR315723), the human *CIA2B* gene (SR309893), or with a universal scrambled siRNA control (SR30004) using Lipofectamine RNAiMAX according to manufacturer’s recommendations. Knockdown was confirmed by Western blot of whole cell lysates and by IF.

### Co-immunoprecipitation

Co-immunoprecipitation was performed using column-based Pierce Direct IP (Thermo Scientific) kit according to manufacturer’s instructions. 300 μL of provided bead slurry was used for each column. 10 μL of each antibody was coupled to the resin using 7 μL of sodium cyanoborohydride. Cells in 10 cm plates were washed in PBS twice before being scraped in lysis-wash buffer. Protein was quantified by BCA assay (Thermo). Seven hundred micrograms of total protein were used for each column. CoIP columns were incubated with gentle rocking at 4 **°**C overnight. Following washing and elution, eluates were pooled and concentrated with 3 K MWCO spin columns (Millipore) prior to gel electrophoresis and Western blot analysis.

### Western blot analysis

Cell extracts were lysed in RIPA buffer and protein concentration determined by BCA assay. 1-5ug total protein samples were reduced in LDS sample buffer prior to separation on 10% bis-tris polyacrylamide gels (Invitrogen) and transfer to PVDF membranes for Western blot (Millipore EMD). Following incubation with chemiluminescent secondary antibodies, blots were scanned and quantitated using an Odyssey CLX imager and associated software. For nuclear cyclin analysis, nuclear lysates were prepared using Pierce NE-PER^TM^ Nuclear and Cytoplasmic Extract reagents (Thermo Scientific) and manufacturer’s recommendations. 3ug of HeLa cell nuclear extract were loaded per lane, and blotted with antibody to either Cyclin E or Cyclin B1 and to nucleolin as a loading control.

### Cell cycle profiling

48 hours following transfection of HeLa cells with either scrambled control siRNA or anti-*Ccdc117* siRNA, cells were harvested by trypsinization, fixed overnight in 70% EtOH at −20 °C, resuspended in PBS, and treated with 1 mg/ml RNAseA (Sigma)/0.05 mg/ml propidium iodide (Invitrogen)/0.3% Triton X-100 at RT for 45 mins in the dark. Stained cells were rinsed in PBS, mesh filtered, and analyzed on a FACS/Aria flow cytometer.

### EdU labeling assay

DNA synthesis rates were determined using the Click-It EdU flow cytrometry assay (Invitrogen). HeLa cells were seeded in 6 well plates at an initial density of 1 × 10^6^ cells/well, then transfected overnight with control scrambled or anti-*Ccdc117* siRNA. Transfected cells were synchronized in G1 by treatment with 20μM Lovastatin 24 hours. Cells were then incubated in fresh medium in the presence of 20uM EdU for 1–6 hours. Following fixing, permeabilization and labeling reactions, cells were counterstained with propidium iodide and EdU incorporation was determined using a Beckman Coulter MoFlow Astrios EQs sorting flow cytometer. Samples were serially gated to identify bulk cells (FSc vs SSc) and isolate single cells (FSc-W vs FSc-H). Propidium iodide (PI) and AF488-EdU were excited using a 355 nm ultraviolet laser and a 488 nm blue-green laser and detected using 620/29 nm and 513/26 nm band pass filters, respectively. Fluorescence was visualized on both univariate and bivariate plots. Cells positive for AF488 were designated as S-phase cells. Total EdU fluorescence was measured on the AF488 histogram. Data were acquired using Summit v6.3 (Beckman Coulter, Fort Collins, CO).

Primer sequences used for mRNA expression qPCR, ChIP qPCR, amplification of *Ccdc117* genomic flanking regions for luciferase reporter constructs are provided in Supplementary Materials, as are details regarding the primary antibodies and dilutions used for ChIP, IF and Western blot experiments.

## Supplementary information

<b>Supplementary Data</b>
