# Supplementary material for: Nkx2–5 Second Heart Field Target Gene Ccdc117 Regulates DNA Metabolism and Proliferation
Source: Sci Rep. 2019 Feb 11;9:1738. doi: 10.1038/s41598-019-39078-5 (PMC6370788; doi:10.1038/s41598-019-39078-5)
Supplement: Supplementary file 1 — Supplementary Data [file 41598_2019_39078_MOESM1_ESM.pdf]

Supplementary Data

Nkx2-5 Second Heart Field Target Gene *Ccdc117* Regulates DNA Metabolism and Proliferation

Anthony J. Horton[1], John Brooker[1], William S. Streitfeld[1], Meaghan E. Flessa[1], Balakrishnan Pillai[1], Raychel Simpson[1,] Christopher D. Clark[1], Monika B. Gooz[3], Kimberly K. Sutton[1], Ann C. Foley[2,4], and Kyu-Ho Lee[1,2,4]*

[1]Departments of Pediatrics and Obstetrics and Gynecology, Medical University of South Carolina, Charleston, SC 29425

[2]Regenerative Medicine and Cell Biology Department, Medical University of South Carolina, Charleston, SC 29425

[3]Dept. of Pharmaceutical and Biomedical Sciences, Medical University of South Carolina, Charleston, SC 29425

[4]Bioengineering Department, Clemson University – MUSC, Charleston, SC 29425

*Corresponding author:

Cardiovascular Developmental Biology Center
Medical University of South Carolina
BSB-601, MSC 508
173 Ashley Avenue
Charleston, SC 29425
Tel.: (843) 792-0307
Email: leekh@musc.edu

Supplemental Figure 1A

A

```
HUMAN   MAALGRPFSG----LPLSGGSDFLQ----------------------PPQPAFPGRAF--------------------PPGADGAELAP------
MOUSE   MAALGRPFSG----LPLSGSADFLQ----------------------PP-PAFAGRAF--------------------PPGAAGHDLAP------
CHICK   MAALGRSCQG----IPAGPALEFPQAAA------------------APDRPVVPGDAVGCVQMVGNGSDGGCGSSGVCPPG--GFDIASLAALGS
FROG    MAAVGTPFNGMLYRMDFHKSSDSLHQISNLQSSIDNSFFSADMSKHNPANPASTGSSV-----NFNNAYFNMYSLCNQSPSSTGGGITPF-----
ZFISH   -MQSSRSGTSELDFIMFSPHAHFSN----------------------PDLVPVSG----------------------------------------

HUMAN   ------RPGPRAVPSSPAG---SAARGRVSVHCKKKHKREEEEDDDCPVRKKRITEAE---LCAGPN------DWILCA--HQDVEGHGVNPSVS
MOUSE   ------RPGVRGAPSSPGG---RTARGRVSIHCRKKHKRLAEDDE-CPVRKKRLTEAE---LGAVTD------EWALGA--HQGREGHGVNTCPS
CHICK   YNEGVAVPPVGSFTPAPHP---QQSHSRVSVRCGKKHKLEEESEG-CPVKKKRLTGAKNCPLNPSTE------EWILCA--GQQAAGE-VATSQY
FROG    ------MPNAIVSPNSGGGLGCSATGSGFPHSSRNKHKREEDIFE-CPLKKRRISEHAEVPIFPEPTTCHSSADILIGETLGSSWDSNCKSKTTT
ZFISH   --------PTKGLHHLTGG---SMPNTSWERRCMRKQRRRTDDES-CSPKRRKLMGEEGANQCPSPKASH---IWPV----DSPAPSPSSETSLV

HUMAN   GLSI-PG--ILDVI----CEEMDQTTGE---PQCEVARRKLQEIEDRIIDEDEEVEADRNVNHL---PSLVLSDTMKTGLKREFDEVFTKKMIES
MOUSE   SLSM-PS--MLDVV----CEEMDQTTGE---PQCEVARRRLQEIEDRIIDEDEEVESDRNVSHL---PSLVLSDTMKTGLKREFDEVFTKRMIES
CHICK   GGSH-PETAMLEIP----CEEMEQTMGE---QQCEVARRKLQEIEDRIIDEDEEVHADGNVSNL---PTLILSDTLKKGMKRDFGEVLTKKIIES
FROG    ALQY-SDSELLPVMHSAQTEEMEESSSESFSPVCGRAQVNY----SRLSSTNDELEMDKTSDHL---PSLIMSDVLKEGLKRGFEESLTKKIVDS
ZFISH   QLQTRPGMEEINLPFTAAPTPLPRVPSEGSGMEVEAAQRRLQEIEERITLEDDSDEEELDVEPAQRRPVLVMSDSLREGLQRGIGDILPHMVAQS

HUMAN   MSRPSMELVLWKPLPELLSDKPKPSSNTKNYTGES----QAKHVAAGTAFPQRTELFSEPR-PTGMSL--YNSLETATSTEEEMEL
MOUSE   MSRPSMELVLWKPLPELLPEKPKPSSSPKNYRRES----QAKHAAPGTAFPQRTEGLLEPQ-CADAPL--YRSLEAATSTEEEMEL
CHICK   MSRPSMELVLWKPLPEFLTDKLKPVS-VKNFRQQSTEGCQAKQSTPRAAFDPQTETFPESQ-QTAMSPDPYPSLGISGCAEEEMEL
FROG    MNRPSMELVLWKPQSELLFDRLQAV--FKSHKKERDLKKPAPSASQTTSFIQEIEIIEDDHLCSSEPTSDLNRTWSRDDEEEEMEL
ZFISH   VSRSCMELVVWRPPEDPLTQRLKDSL-QRQQRKSR ----QTPTPVPSVSSPPSHQQTFSPL-------------FSNSGEEDMEL
```

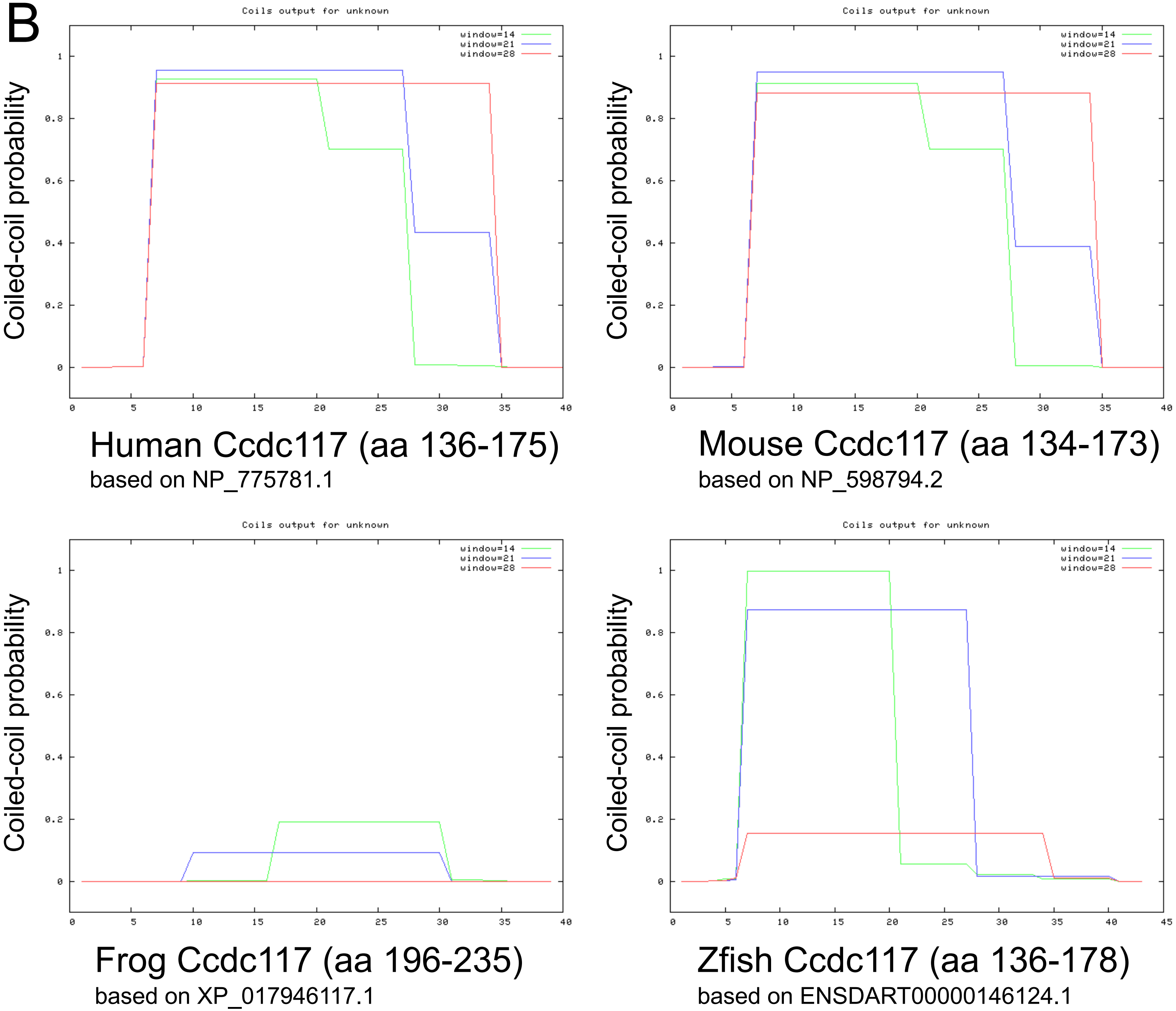

Supplemental Figure 1B

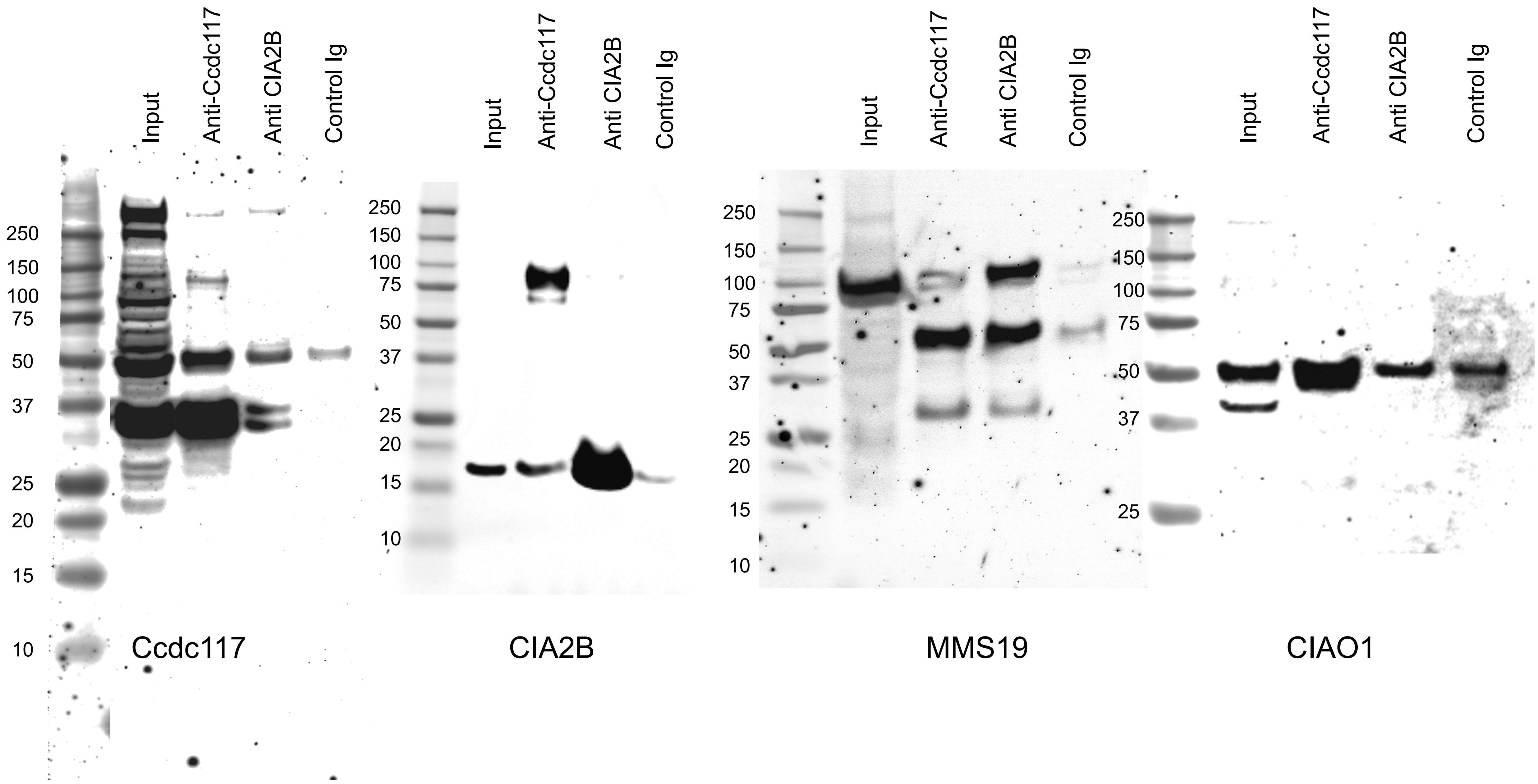

Supplemental Figure 2A

Supplemental Figure 2B

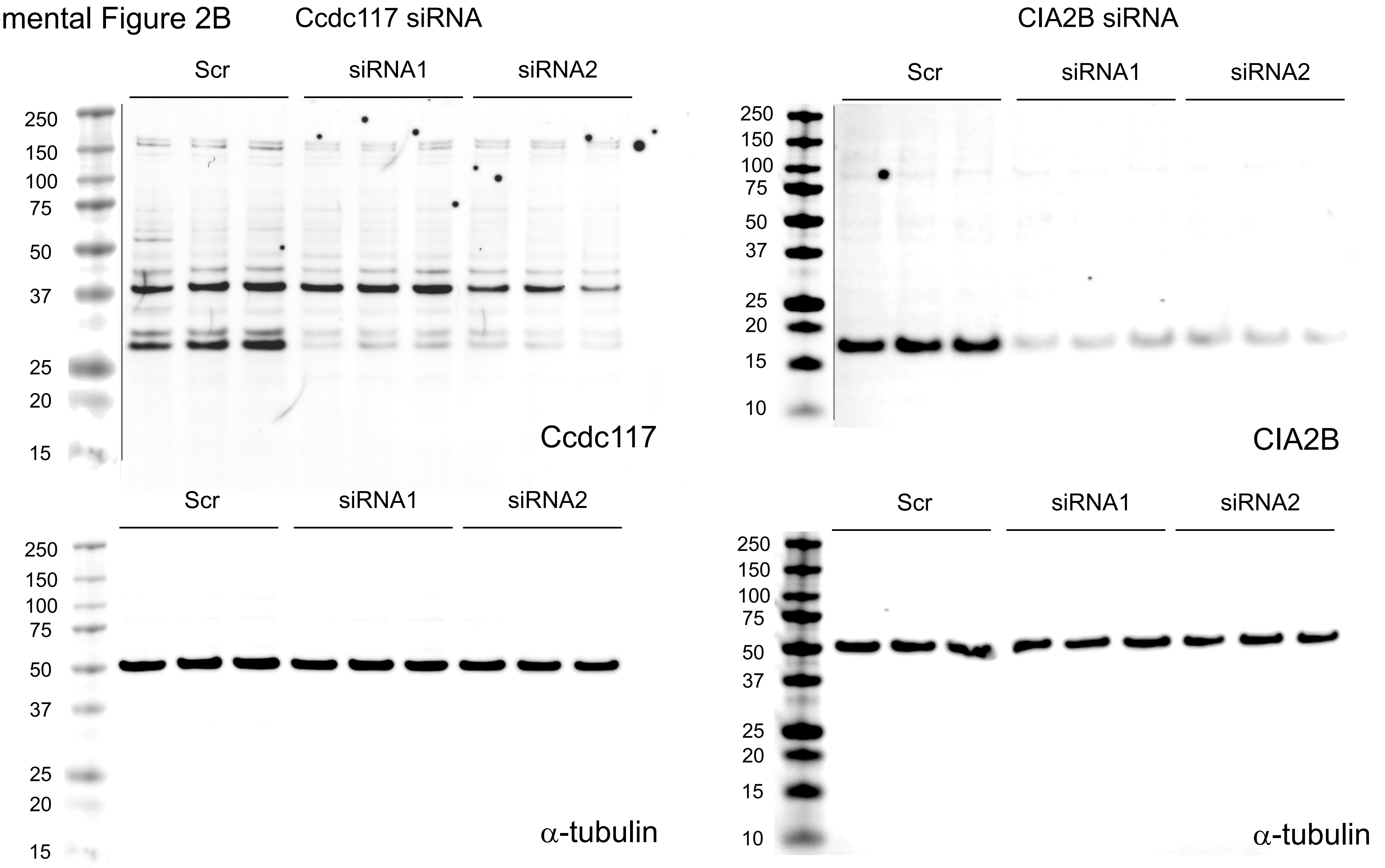

MMS19

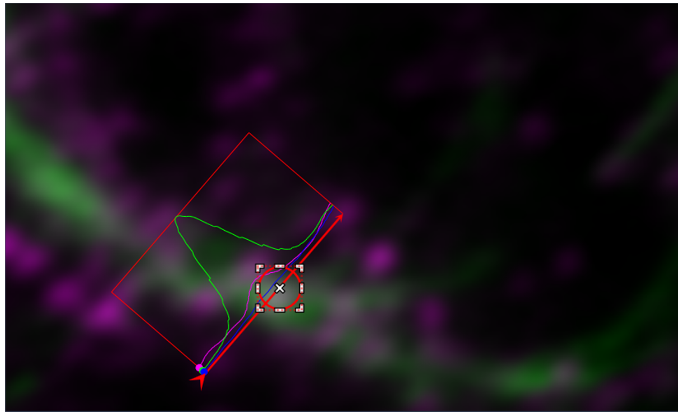

Ccdc117

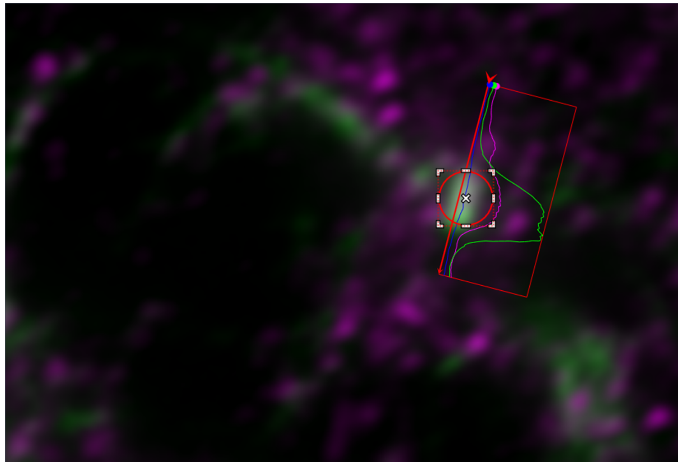

CIA2B

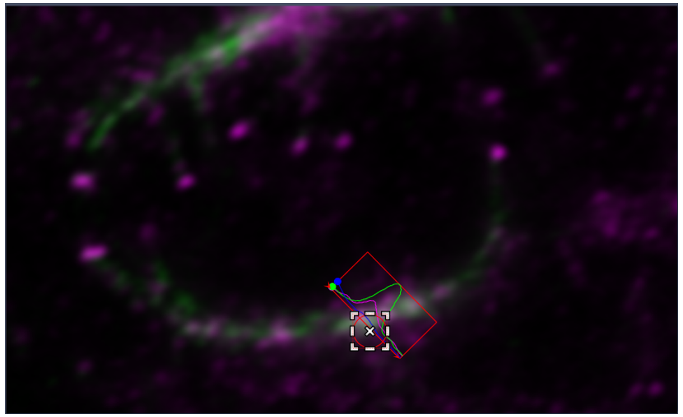

CIAO1

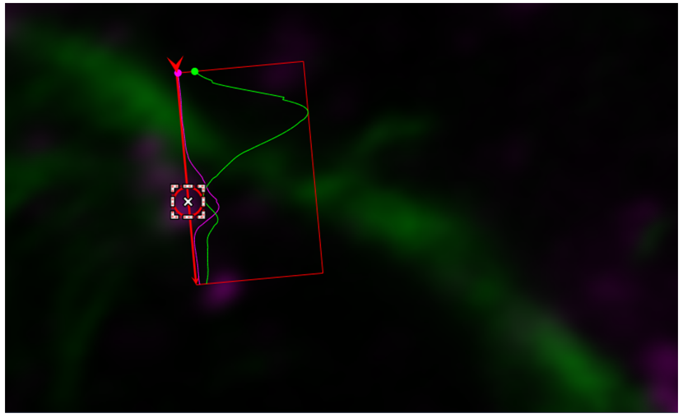

Zen calculated Pearson's coefficient (0-1 max)	
Ccdc117 v α-tubulin	0.85937
MMS19 v α-tubulin	0.91394
CIA2B v α-tubulin	0.94000
CIAO1 v α-tubulin	0.73806

Supplemental Figure 3

Supplementary Table 1: Primers

**qPCR Primers**	**Forward**	**Reverse**
Ccdc117	TGT TAG CCA CCT CCC AAG TC	GAG GTT TCC AGA GCA CAA GC
B actin	CGG GAC CTG ACA GAC TAC CTC	AAC CGC TCG TTG CCA ATA

**Primers for Constructs**	**Forward**	**Reverse**
Ccdc117 promoter	GCT Gcc atg gCT TTC CAG	ggc ctc gag TAG AAT AAT AAC TCC AGC TGT CAG GTC
Ccdc117/Xbp1 3' enhancer	ggc aga tct AGC GTC CTG TTC TGT GAT TTC	ggc aga tct TTT AAA GTA TCT GTA ATC CTG TAT AAA TGT AA

**qPCR primers for ChIP**	**Forward**	**Reverse**
Ccdc117 promoter	GCT GTC AGG TCC CAC TCC TA	GGC AGA GGA GGG AAA ATC TC
Region A	ACC AAC CTG ACA CCA ACA CA	CAC TGC ACC CTA ATT GCT GA
Region B	AGG GGA CCA AGA GAA CCA AC	TGT CAT TCC ATC ACC CAA GA
Region C	CAG TGA GCT TCA GGG CTC TT	GTT TAG GGG GAT GGG TCA GT
Region D	AGG CCA AGT TCC AGT GTT GT	GCC ACT TCA ATG CAA TGC TA
Region E	CCA CCT GTG CTC TGG AAA GT	TCC ATT CCA GAG ATG GAA CC
Region F	ACA CAG CGT CCT GTT CTG TG	CCC CAA AAG GTG GTT AGG TT

Supplementary Table 2: Antibodies

Antigen	Dilution	Source	Catalog reference
Ccdc117	1:10,000 (western blot)<br>1:200 (IF)	Sigma	HPA000826
CIA2B	1:10,000 (western blot)<br>1:100 (IF)	Abcam	ab166607
MMS19	1:10,000 (Western blot)<br>1:100 (IF)	Abcam	ab188156
CIAO1	1:500 (Western blot)<br>1:100 (IF)	Abcam	ab83088
alpha Tubulin	1:10,000 (Western blot)<br>1:100 (IF)	Sigma	T7451
Cyclin B1	1:800 (Western blot)	Santa Cruz	sc-245
Cyclin E	1:800 (Western blot)	Santa Cruz	sc-247
Nucleolin	1:4000 (Western blot)	Novus	NB600-241SS
gamma-phospho-histone H2AX (Ser139)	1:100 (IF)	EMD Millipore	05-636-I
phospho-ATM (Ser 1981)	1:100 (IF)	eBioscience	14-9046-82
phospho-histone H3 (Ser 10)	1:100 (IF)	Cell Signaling	9701S
activated Caspase 3	1:100 (IF)	Cell Signaling	9661S

Supplemental Figure 1. Conservation of Ccdc117 in vertebrates. **A**: Protein sequence alignment of representative mammalian, avian, amphibian and zebrafish vertebrate homologs of *Ccdc117* by Clustal W analysis. Red boxed region indicates predicted coiled-coil domain. **B.** Ccdc117 sequence analysis using the COILS program [48].

Supplemental Figure 2. Full length Western blots for **A**. co-IP experiments and **B**. siRNA knockdown of Ccdc117 and CIA2B

Supplemental Figure 3: Supplementary figure shows overlays of signal overlap histogram curves on actual Z-stack images showing highest degrees of co-localization. The high Pearson correlation coefficient (~1) shows high degree of co-localization of the two proteins.

Details for M-phase IF co-localization measurements. For statistical quantification of co-localization ratios of the two fluorophores we calculated the Pearson correlation coefficient (REF) using the Zen software (Carl Zeiss MicroImaging, LLC, Thornwood, NY).

Reference: Adler J1, Parmryd I. Quantifying colocalization by correlation: the Pearson correlation coefficient is superior to the Mander's overlap coefficient. Cytometry A. 2010 Aug;77(8):733-42.

Supplementary Table 1. Oligonucleotide sequences for cloning, qPCR and in vivo ChIP qPCR experiments.

Supplementary Table 2. Antibody sources and dilutions used for IF and Western blot experiments.
